# Тестостеронзаместительная терапия – исторический экскурс. Почему не каждый лабораторный результат совпадает с клиникой? Ожидаемый побочный эффект данной терапии.

**DOI:** 10.14341/probl12742

**Published:** 2021-12-03

**Authors:** И. И. Голодников, З. Ш. Павлова, А. А. Камалов

**Affiliations:** Российская медицинская академия непрерывного профессионального образования Минздрава России; Медицинский научно-образовательный центр МГУ им. М.В. Ломоносова; Медицинский научно-образовательный центр МГУ им. М.В. Ломоносова

**Keywords:** тестостерон, эстрадиол, эстрогены

## Abstract

Тестостеронзаместительная терапия (ТЗТ) сегодня является одним из наиболее эффективных и распространенных методов лечения дефицита тестостерона. Ее часто назначают, ориентируясь только на уровень тестостерона и наличие жалоб пациента на снижение половой функции. Довольно редко специалисты дополнительно оценивают уровень эстрадиола и, как следствие, изначальное наличие симптома гиперэстрогении. Одним из вероятных последствий назначения ТЗТ как раз и может стать избыток эстрадиола, избыточное образование которого связано с ферментом ароматазой, конвертирующей тестостерон в эстрадиол. Несмотря на доступность лабораторного определения уровня эстрадиола, результат не всегда может совпадать с клинической картиной, одной из причин является различие в методиках определения уровня эстрадиола в каждой лаборатории, единый стандарт, или «эталон», на сегодня отсутствует. В данной статье приведено описание эволюции ТЗТ, а также сделан акцент на вариабельность уровня эстрадиола от лаборатории к лаборатории, подробно объяснено, почему оценка эстрадиола в динамике должна проводиться только в одной лаборатории. Системный поиск литературы проводился по базам данных Medline, Scopus, Web of Science и Elibrary, КиберЛенинка.

Целью нашего обзора стала необходимость привлечь внимание специалистов к проблеме гиперэстрогении, не всегда оправданного назначения ТЗТ у пациентов с ожирением, гипогонадизмом и гиперэстрогенией, а также к проблеме лабораторной диагностики гиперэстрогении, остро стоящей не только в нашей стране, а во всем мире.

## Abstract (en)

Testosterone replacement therapy (TRT) is one of the most effective and common treatments for testosterone deficiency today. It is often prescribed, focusing only on the level of testosterone and the presence of patient complaints about a decrease in sexual function. Quite rarely, doctors additionally assess the level of estradiol and, as a consequence, the initial presence of a symptom of hyperestrogenism. One of the likely consequences of the appointment of TRT may be an excess of estradiol, the excess formation of which is associated with the enzyme aromatase, which converts testosterone into estradiol. Despite the availability of laboratory determination of the level of estradiol, the result may not always coincide with the clinical picture, one of the reasons is the difference in the methods for determining the level of estradiol in each laboratory, there is no single standard or «reference» today. This article describes the evolution of TRT, and also focuses on the variability of estradiol levels from laboratory to laboratory, and explains in detail why the assessment of estradiol over time should be carried out in only one laboratory. A systematic literature search was carried out in the databases Medline, Scopus, Web of Science and Elibrary, CyberLeninka.

The purpose of our review was the need to engage the attention of specialists to the problem of hyperestrogenism, the not always justified prescription of TRT in patients with obesity, hypogonadism and hyperestrogenism, as well as to the problem of laboratory diagnosis of hyperestrogenism, which is acute not only in our country, but all over the world.

## ВВЕДЕНИЕ

Ожирение, кроме системного субклинического воспаления жировой ткани [1], сопровождается коморбидными состояниями. И одно из самых распространенных и опасных таких состояний у мужчин — это дефицит тестостерона. Согласно исследованиям российских ученых, у мужчин старше 45 лет распространенность дефицита тестостерона составляет от 10 до 40% [2]. И большую лепту в эту статистику вносят случаи с избыточной массой тела или ожирением. Количество мужчин с лишним весом прогрессирует во всем мире, и вместе с ним прогрессирует и проблема андрогенного дефицита. В Европейском исследовании старения мужчин (EMAS), участниками которого были 3369 мужчин в возрасте 40–79 лет (средний возраст 60 лет), было отмечено, что мужчины с ожирением имели уровень общего тестостерона на 30% ниже (на 5,1 нмоль/л), и на 18% ниже был свободный тестостерон (на 53,7 пмоль/л) по сравнению с теми мужчинами, индекс массы тела (ИМТ) которых был меньше ≤25 кг/м2, то есть в норме. В EMAS ожирение было связано с 13-кратным увеличением риска развития гипогонадизма, в то время как наличие 2 или более сопутствующих заболеваний увеличивает риск развития дефицита тестостерона только в 5,2 раза [3][4]. То есть есть существенная разница между тем, имеют ли мужчины коморбидные заболевания или нет. При отсутствии сопутствующих патологий гипогонадизм встречается в общей популяции мужчин примерно в 5% случаев [5]. При наличии таких заболеваний, как ожирение, особенно его висцеральная форма или сахарный диабет 2 типа, встречаемость гипогонадизма резко повышается, по данным других авторов, до 30% [6][7].

Второй важный фактор увеличения количества дефицита тестостерона — увеличение продолжительности жизни. Научно-технические достижения в медицине повысили шансы многих мужчин дожить до старости и, следовательно, до возможности истощения эндокринной функции тестикулярной ткани. Иначе говоря, до истинного дефицита тестостерона. По имеющимся у нас данным, за последние 100 лет в масштабах всего мира количество мужчин, доживающих до 65 лет, увеличилось в 7 раз, а тех, кто преодолел возраст 85 лет, стало больше в 31 раз [8].

Поэтому сегодня актуальность изучения самого андрогенного дефицита и методов его коррекции высока, как никогда раньше.

## ИСТОРИЧЕСКИЙ ЭКСКУРС

Самым очевидным и наиболее эффективным средством компенсации дефицита тестостерона является тестостеронзаместительная терапия (ТЗТ) с использованием разных лекарственных форм тестостерона.

Сейчас ТЗТ никого не удивишь, это рабочий инструмент врачей с тех пор, как в 40-х годах прошлого века впервые были синтезированы половые гормоны. А первые опыты заместительной терапии для стареющего человеческого организма были проведены еще в конце XIX в., и эти попытки были малоэффективны и далеко не безобидны.

Одним из ярких примеров научного поиска в этой области был Шарль Броун-Секар, французский врач и ученый, который экспериментировал не только на животных, но и на себе. Он впрыскивал себе под кожу водный настой яичек морских свинок и собак и уверял, что, несмотря на заметную боль в месте инъекции, он ощущает повышение общего тонуса, мышечной силы и интеллектуальных возможностей, улучшение работы кишечника и органов малого таза. Свои опыты он описал в научном журнале в 1889 г., что привело к множеству подражателей. Последователи также описывали подобные эффекты и даже внесли свой вклад в науку, описав период падения тонуса и всего остального после окончания действия инъекций.

Надо отметить, что Броун-Секар начал свои поиски в отношении заместительной гормональной терапии именно с наблюдения за животными. Как известно, период максимальной активности и здоровья у любого организма совпадает с активным репродуктивным периодом. Из чего французский ученый, как и многие другие, сделал вывод, что в ткани яичек вырабатывается некое вещество, которое «омолаживает» весь организм.

В тот период времени еще не было такого понятия, как «гормон». Впервые этот термин появился в 1902 г. в работах английских физиологов У. Бейлисса и Э. Старлинга. В переводе с древнегреческого «гормон» (ὁρμάω) означает «двигаю, побуждаю».

В 1935 г. Э. Локьер впервые выделил из бычьих тестикул «мужской гормон» — тестостерон. В этом же году немецкий ученый Адольф Бутенандт не только выделил, но и описал структуру тестостерона. А через неделю югославский ученый Леопольд Ружечка осуществил частичный синтез тестостерона из холестерина. За эти исследования Бутенандт и Ружечка были удостоены Нобелевской премии по физиологии и медицине.

Достаточно долгое время ТЗТ использовалась при особых случаях, например, при анорхизме [9]. Вероятнее всего, это было связано с работами Huggens и Hodges в 1941 г., за которые они в последующем получили Нобелевскую премию по физиологии и медицине. Суть работы заключалась в том, что кастрация (то есть практически полное прекращение синтеза тестостерона) эффективна при диссеминированном раке предстательной железы.

С высокой долей вероятности этим и было определено достаточно долгое, практически до 1990-х годов, уменьшение интереса к медицинскому использованию ТЗТ. Иначе говоря, если уменьшение уровня собственного тестостерона оказывало благотворный эффект, то кто бы стал использовать экзогенные формы тестостерона для мужчин.

В то же время в бодибилдинге и профессиональном спорте интерес к этому гормону только рос, несмотря на все опасения и запреты со стороны официальной медицины.

Дело усугубляло то, что периодически просачивалась негативная информация в отношении ТЗТ. Например, в конце 2013 и в начале 2014 гг. в американской прессе, в уважаемых изданиях, появились результаты ретроспективного исследования о негативном влиянии ТЗТ на сердечно-сосудистую систему [10][11]. Резонанс достиг такого уровня, что американская организация FDA, регулирующая пищевые продукты и лекарственные средства, внесла существенные ограничения для ТЗТ, обязав производителей указывать наличие повышенных сердечно-сосудистых рисков [12]. Также назначение ТЗТ должно быть только в случаях обоснованных причин дефицита тестостерона, причем возрастной гипогонадизм, или естественное снижение уровня половых гормонов, перестало входить в их число [13].

Таким настроениям подверглись не все страны и организации. Например, Европейское Агентство по лекарственным средствам (ЕМА) не стало вносить никаких ограничений на основании представленных данных [14].

Вся эта негативная информация оказала свое действие, и многие врачи, как и многие пациенты, стали отказываться от назначения или применения ТЗТ, опасаясь тяжелых побочных эффектов, прежде всего со стороны сердечно-сосудистой системы.

Но уже совсем скоро, в 2016 г., были опубликованы результаты большого рандомизированного клинического исследования, в котором продемонстрированы положительные эффекты ТЗТ у мужчин по показаниям, не связанным с классическим гипогонадизмом. Например, андрогенный дефицит, который развивается на фоне дефектов гипоталамо-гипофизарно-гонадной оси. Это способствовало началу восстановления доверия общества к обоснованно назначенной ТЗТ [15]. Кроме того, существует большое количество работ, которые демонстрируют высокую эффективность и безопасность ТЗТ при ожирении, которое способствует развитию всех компонентов метаболического синдрома, сердечно-сосудистых патологий, инсулинорезистентности, сахарного диабета (СД) 2 типа и прочих метаболических патологий [3][4][16][17].

## ВЗАИМОСВЯЗЬ ТЕСТОСТЕРОНА И ЭСТРАДИОЛА

Несмотря на то что теперь ТЗТ проводится лекарственными средствами, безопасность и эффективность которых подтверждены многочисленными исследованиями [18–20], она не должна назначаться любому пациенту, у которого обнаружен сниженный уровень тестостерона, тем более, однократно определенный, в том числе и на фоне ожирения. Кроме того, на фоне статей, подтверждающих эффективность ТЗТ, есть убедительные работы, которые акцентируют внимание на негативных сторонах ТЗТ и обосновывают свою позицию [21, 22]. Прежде всего важно модифицировать образ жизни и мотивировать пациента к снижению объема жировой ткани, так как без этих важнейших действий можно не добиться никакого эффекта даже при использовании максимально успешных препаратов [23]. Тестостерон не является конечной точкой в стероидогенезе. После него как минимум два гормона достойны внимания и контроля, особенно гормон эстрадиол как наиболее активная форма эстрогенов (рис. 1) [24].

**Figure:**
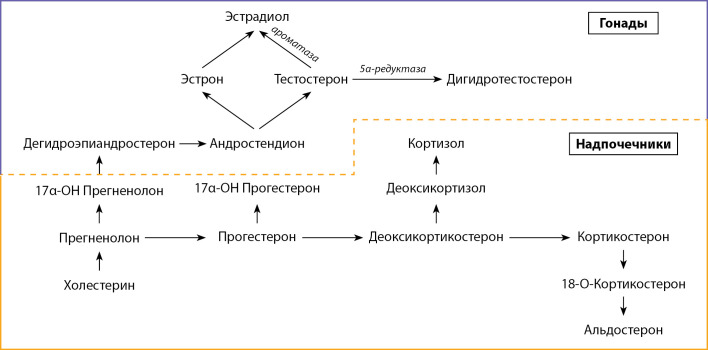
Рисунок 1. Схема стероидогенеза.

Если ориентироваться на клинические рекомендации, то мы можем контролировать только уровень тестостерона и при первичном обследовании — уровень лютеинизирующего гормона (ЛГ) [25]. Но если апеллировать к стероидогенезу (см. рис. 1) и патогенезу ожирения, то контроль уровня эстрадиола кажется очевидным. Из имеющихся на сегодняшний день рекомендаций по диагностике гипогонадизма мы можем опираться на сбор жалоб и анамнеза в отношении двух основных признаков: снижение либидо и ухудшение эректильной функции (уровень убедительности рекомендаций В; уровень достоверности доказательств 3) [4][5]. При этом важно исключить сопутствующие заболевания, прием лекарственных или наркотических средств (уровень убедительности рекомендаций В; уровень достоверности доказательств 3) [26][27]. Также важно провести физикальное обследование с определением окружности талии, оценить рост волос на теле, органы мошонки, половой член, предстательную железу и грудные железы (уровень убедительности рекомендаций В; уровень достоверности доказательств 3) [28–33]. И важнейшим диагностическим инструментом в диагностике дефицита тестостерона является лабораторная диагностика. Для разграничения нормального состояния и потенциального дефицита тестостерона нужно использовать уровень общего тестостерона в сыворотке крови 12,1 нмоль/л при заборе крови натощак в утренние часы, между 7 и 11 ч (уровень убедительности рекомендаций А; уровень достоверности доказательств 2) [34][35]. При уровне общего тестостерона в диапазоне от 8 до 12 нмоль/л рекомендовано определение уровня глобулина, связывающего половые стероиды, для определения свободного тестостерона, нижний референс которого составляет 243 пмоль/л (уровень убедительности рекомендаций А; уровень достоверности доказательств 2) [33][34].

На сегодняшний день рекомендации по контролю эстрадиола у мужчин, к сожалению, отсутствуют, но мы не теряем надежды, что они появятся, потому что упомянутое ранее ожирение способствует развитию инсулинорезистентности, а затем и повышению активности фермента ароматазы (CYP19A1) [36][37]. А это, в свою очередь, будет избыточно конвертировать тестостерон в эстрадиол, уменьшая уровень тестостерона и повышая уровень эстрадиола [38][39].

Если врач оценит только уровень тестостерона, без определения исходного уровня эстрадиола, назначение ТЗТ может быть ошибочным.

Эстрадиол разумно оценивать и во время проведения ТЗТ, так как достижение супрафизиологических уровней тестостерона будет усиливать активность ароматазы и, соответственно, вести к росту эстрадиола.

Если вернуться к резонансным публикациям 2013–2014 гг., то описанные результаты ретроспективного исследования, вполне возможно, как раз и были связаны со сформировавшейся гиперэстрогенией на фоне ТЗТ и, как следствие, возникшей повышенной частотой сердечно-сосудистых событий. Теперь пролить свет на те события будет сложнее, потому что мы не знаем, проводился ли контроль эстрадиола у тех пациентов и возможно ли сопоставление исходных уровней эстрадиола и уровней его на фоне ТЗТ.

Негативные последствия гиперэстрогении мы детально описали в предыдущих статьях [40][41] и считаем возможным только вкратце упомянуть грозные осложнения гиперэстрогении, в том числе подтверждающие наши предположения в отношении повышения сердечно-сосудистых рисков на фоне ТЗТ. Большое количество специалистов рассматривают гиперэстрогению как фактор, повышающий риск смерти у пациентов с сердечной недостаточностью, маркер онкологических заболеваний и состояние, повышающее риски развития опухолей разной локализации [42–44]. Кроме того, одним из ярких клинических проявлений гиперэстрогении, в том числе и на фоне ТЗТ, является гинекомастия. По мнению американских ученых, гинекомастия у взрослых и молодых людей в количестве от 1/3 до 2/3 случаев прогрессирует на фоне ТЗТ, когда в крови создаются супрафизиологические уровни тестостерона и повышается активность ароматазы. И те же явления уже в 70% случаев развиваются у мужчин в возрасте от 50 до 69 лет [29]. Гинекомастия — это доброкачественная пролиферация железистой ткани грудной железы у мужчин. Это определение касается истинной гинекомастии. В отличие от ложной гинекомастии, грудной железистой ткани, при избыточно развитой только жировой ткани в области грудных мышц, нет. Частота развития гинекомастии очень высокая, от 32 до 65%, в зависимости от разных факторов и критериев, в том числе и от возраста [45]. Также одно из исследований, результаты которого были опубликованы в 2018 г., продемонстрировало, что ТЗТ повышает активность ароматазы и экспрессию эстрогенных и андрогенных рецепторов, что способствует нарушению баланса андрогенов и эстрогенов в пользу последних [46].

Если в среде российских исследователей проблема гиперэстрогении обсуждается крайне осторожно и редко, то в других странах этой проблеме уделяют большее внимание, в том числе и в оценке нормального, физиологичного уровня эстрадиола у здоровых мужчин. Например, в одной из работ американских исследователей за 2019 г. были представлены данные по изменению уровня эстрадиола у мужчин в Америке, в масштабах страны (использовали данные 3-го национального исследования здоровья и питания (NHANES III; 1988–1991) и непрерывного NHANES (1999–2004)) в разных возрастных группах, с указанием того, что мужчины без ожирения, не курящие, без сердечно-сосудистых и онкологических заболеваний, также без диабета, и эти уровни эстрадиола показательно уменьшаются с ростом количества лет [29]. Это логично, так как снижается синтез тестостерона как источник эстрадиола. Но если ориентироваться на ту часть мужского населения с избыточно развитой жировой тканью и/или ожирением, которая уже превалирует в обществе, то мы можем предположить, как существенно повышается уровень эстрадиола у мужчин с нарушенным составом тела, так как активность ароматазы прогрессивно увеличивается на фоне каскада цитокинов, продуцируемых в избыточно развитой жировой ткани. К сожалению, пока распространенность гиперэстрогении принято оценивать по распространенности гинекомастии. Еще одним примером высокого интереса к изменениям уровней эстрадиола в целом и на фоне ТЗТ в частности является исследование, которое было опубликовано в феврале 2020 г. [47]. Ученые давали оценку тому, как врачи контролируют уровень эстрогенов при проведении ТЗТ у своих пациентов. С учетом того, что эта терапия становится все распространеннее, актуальность контроля уровня эстрогенов также повышается.

Исследование проводилось методом анонимного опроса в электронном виде среди членов Международного общества сексуальной медицины, то есть среди врачей, которые используют в своей практике ТЗТ. Кроме вопросов о контроле эстрогенов, исследователи ставили вопросы о симптомах гиперэстрогении, антропометрических данных и подходах к лечению гиперэстрогении на фоне ТЗТ.

Результаты оказались неожиданными:

Надо оговориться, что ингибиторы ароматазы в США официально используются не только у женщин при раке молочной железы, но и у мальчиков в подростковом периоде при наличии истинной гинекомастии. Так вот, 47,7% врачей назначали эти препараты при бессимптомном течении лабораторно подтвержденной гиперэстрогении.

И совершенно неожиданным был результат, что в 14,4% случаев респонденты назначали препараты из класса ингибиторов ароматазы в профилактических целях, предполагая развитие гиперэстрогении у этих пациентов как закономерное явление при достижении супрафизиологических значений уровня тестостерона.

Врачи, которые занимались не только практической деятельностью, но и научной, чаще использовали ингибиторы ароматазы и при наличии симптомов гиперэстрогении, и в профилактических целях.

Какие выводы сделали исследователи?

Во-первых, большая часть практикующих врачей (более 62%, использующих ТЗТ) контролируют уровень эстрогенов.

Во-вторых, критерием для назначения терапии могут быть симптомы гиперэстрогении.

В-третьих, отмечается большая вариабельность в схемах назначений ингибиторов ароматазы.

Надо отметить, что пока небольшое количество российских ученых, так же как и наших зарубежных коллег, проблема гиперэстрогении волнует, и уже не первый год, несмотря на то, что тема недостаточно освещена. Примером тому служит исследование, результаты которого были изложены в диссертационной работе в 2019 г., посвященной особенностям диагностики и лечения бесплодия у мужчин с ожирением и избыточной массой тела [45]. В данной работе было выявлено, что распространенность мужчин с бесплодием и избыточной массой тела составляет 36,8%, а с ожирением — 16,6%. И у таких мужчин нарушения сперматогенеза чаще всего связаны с наличием полиморфизмов rs2414096 и rs749292 гена ароматазы CYP19A1, которые способствуют развитию гиперэстрогении. В этой же работе исследователи продемонстрировали высокую эффективность ингибиторов ароматазы не только в увеличении уровня тестостерона и снижении избыточного уровня эстрадиола, но и существенного улучшения спермограммы у таких пациентов в сравнении с применением тамоксифена [45].

И, конечно же, врачи, которые проводят контроль уровня эстрогенов на фоне ТЗТ, ищут способы эффективной и безопасной борьбы с гиперэстрогенией у мужчин, помогают формированию опыта для дальнейшего понимания влияния низких и высоких уровней эстрогенов и формирования стандартов терапии [47].

В этом исследовании не ставился вопрос о длительности используемых схем назначения препаратов, препятствующих развитию последствий гиперэстрогении и профилактики дальнейшего ее развития. А это один из актуальнейших моментов.

Какие еще вопросы часто вызывают горячие споры среди клиницистов? Огромное количество дискуссий вызывает лабораторная диагностика, особенно в отношении эстрогенов в крови. У каждого врача формируется свое отношение к той или иной лаборатории. Одни специалисты ориентируются на используемые методы, отдавая предпочтение масс-спектрометрии. Другие предпочитают рутинные иммунологические методы или ставят в приоритет стоимость анализов для пациентов.

Мы в своей практике также сталкиваемся с тем, что одна и та же проба крови в разных лабораториях может дать отличающиеся результаты, вплоть до несопоставимых значений. Это может ввести врача в заблуждение и привести к необоснованным назначениям с соответствующими результатами. К тому же это может вызвать сомнения у пациента, и его приверженность к лечению снизится. Увы, это явление характерно для всего мира.

В качестве примера хотим привести результаты одного из актуальных исследований, опубликованного в 2014 г., в связи с прогрессирующим ростом онкологических заболеваний и количества людей с ожирением [48]. Целью исследования была стандартизация измерений эстрадиола. Для оценки точности и вариабельности измерений эстрадиола в сравнении использовали 11 иммунологических методов и 6 методов масс-спектрометрии проб однородной сыворотки с эталонным методом. Учитывали вклад в конечные результаты калибровки, специфичности или матричных эффектов. Чувствительность разных методов существенно отличается. Большинству методов не под силу объективно определять уровень эстрадиола менее 10 пг/мл. Одним из важнейших элементов, вносящих свою лепту в высокую вариабельность при определении уровня эстрадиола, является недостаточная специфичность, особенно при низких концентрациях эстрадиола, что позволило ученым предположить, что несколько отличающиеся от эстрадиола молекулы вносили свой вклад в результат измерения как раз за счет недостаточной специфичности.

Результаты исследования подтвердили озабоченность врачей и продемонстрировали существенную вариабельность эстрадиола в сыворотке крови как мужчин, так и женщин. Среднее смещение по всем образцам для каждой пробы крови отличалось от -2,4% до +235%, при этом у трех участников среднее смещение превышало 100%. В выводах было подчеркнуто, что смещение калибровки является главной причиной общей изменчивости результатов. Стандартизация, то есть калибровка по общему стандарту с использованием панелей отдельных образцов, может снизить широко распространенную во всем мире вариабельность и повысить объективность производимых лабораторных диагностических тестов на эстрадиол. Напомним, что на основании этих результатов анализов врачи принимают решения и назначают терапию, как правило, препаратами, воздействующими на гормональный фон. Иначе говоря, от правильности произведенных анализов зависят здоровье и жизнь пациентов, и ошибок здесь быть не должно.

Кроме того, эти ошибки выливаются в значительные суммы денег, которые тратит государство на компенсацию утраченного гражданами здоровья. Например, когда развитие многих заболеваний можно было предотвратить, используя повышенный уровень эстрадиола как онкомаркер. Ведь ряд эпидемиологических исследований показал, что наличие высокого уровня эстрадиола у женщин в постменопаузе повышает риск развития рака молочной железы от 1,5 до 3 раз [28][35][49–51]. И таких исследований значительно больше, и не только в онкологической сфере. О том, что гиперэстрогения у мужчин также рассматривается как фактор, повышающий риски развития сердечно-сосудистых катастроф или онкологических заболеваний, мы уже упоминали ранее [42–44]. Почему же огромный опыт, который накоплен в отношении женщин, не может быть использован хотя бы как основа для более детального изучения проблемы гиперэстрогении у мужчин?

## ЗАКЛЮЧЕНИЕ

При изложенных выше фактах становится очевидным, почему так мало доступной информации по поводу гиперэстрогении у мужчин, при том, что даже в отсутствие рекомендаций врачи контролируют уровень эстрогенов и используют ингибиторы ароматазы, например, в Америке. Во-первых, недостаточно статей, акцентирующих внимание специалистов и ученых на широкой распространенности гиперэстрогении у мужчин. До сих пор распространенность гиперэстрогении у мужчин оценивается по распространенности гинекомастии. Сложность заключается в том, что гинекомастия может еще не успеть развиться на фоне гиперэстрогении на период обследования, а гиперэстрогения уже может быть. То есть использование гинекомастии как ключевого клинического признака может быть недостаточным. Во-вторых, при отсутствии обозначения актуальности проблемы нет полномасштабных клинических исследований, которые бы доказали или опровергли наличие данной проблемы. В-третьих, соответственно, не может появиться рекомендаций, на которые бы могли опираться врачи в назначении обследования мужчин с андрогенным дефицитом и нарушениями состава тела. В-четвертых, нет полной уверенности в объективности определения уровня эстрадиола в лабораториях, что может способствовать получению ложнозавышенных или ложнозаниженных результатов и отсутствию эффективности лечения. В-пятых, нет достоверных данных о безопасности и эффективности использования различных средств, способствующих снижению уровня эстрогенов или блокирующих их воздействие на эстрогеновые рецепторы.

Иначе говоря, при очевидности проблемы гиперэстрогении недостаточное количество публикаций, содержащих убедительную информацию, высокий уровень убедительности рекомендаций и достоверности доказательств, наличие массы противоречивой информации и сформировало позицию научного сообщества, находящегося в ожидании достоверных данных, которые могут послужить прорывом и новым шагом на пути улучшения здоровья и качества жизни мужчин.

## ДОПОЛНИТЕЛЬНАЯ ИНФОРМАЦИЯ

Источники финансирования. Работа выполнена по инициативе авторов без привлечения финансирования.

Конфликт интересов. Авторы декларируют отсутствие явных и потенциальных конфликтов интересов, связанных с содержанием настоящей статьи.

Участие авторов. Голодников И.И. — вклад по критериям 1(b), 2, 3, 4; Павлова З.Ш. — вклад по критериям 1(а) 2, 3, 4; Камалов А.А. — вклад по критериям 1(а) 2, 3, 4.

Все авторы одобрили финальную версию статьи перед публикацией, выразили согласие нести ответственность за все аспекты работы, подразумевающую надлежащее изучение и решение вопросов, связанных с точностью или добросовестностью любой части работы.
